# Organoids as a Powerful Model for Respiratory Diseases

**DOI:** 10.1155/2020/5847876

**Published:** 2020-03-11

**Authors:** Yu Li, Qi Wu, Xin Sun, Jun Shen, Huaiyong Chen

**Affiliations:** ^1^Department of Basic Medicine, Tianjin University Haihe Hospital, Tianjin, China; ^2^Key Research Laboratory for Infectious Disease Prevention for State Administration of Traditional Chinese Medicine, Tianjin Institute of Respiratory Diseases, Tianjin, China; ^3^Tianjin Key Laboratory of Lung Regenerative Medicine, Tianjin, China

## Abstract

Insults to the alveoli usually lead to inefficient gas exchange or even respiratory failure, which is difficult to model in animal studies. Over the past decade, stem cell-derived self-organizing three-dimensional organoids have emerged as a new avenue to recapitulate respiratory diseases in a dish. Alveolar organoids have improved our understanding of the mechanisms underlying tissue homeostasis and pathological alterations in alveoli. From this perspective, we review the state-of-the-art technology on establishing alveolar organoids from endogenous lung epithelial stem/progenitor cells or pluripotent stem cells, as well as the use of alveolar organoids for the study of respiratory diseases, including idiopathic pulmonary fibrosis, tuberculosis infection, and respiratory virus infection. We also discuss challenges that need to be overcome for future application of alveolar organoids in individualized medicine.

## 1. Introduction

Organoids are stem/progenitor cell-derived three-dimensional (3D) structures within an extracellular matrix that recapitulate essential structural and functional aspects of multiple organs, including the mammary gland, liver, pancreas, tongue, stomach, prostate, and lung [1]. In the lung, damage to the epithelia that cover the conducting airways and alveoli may lead to an inflammatory storm and progressive diseases, including bronchial asthma, chronic obstructive pulmonary disease, and idiopathic pulmonary fibrosis (IPF), as well as respiratory infections such as the recent coronavirus COVID-19 in China [2]. Region-specific stem/progenitors have been characterized for the maintenance of lung epithelia or to repair the lung epithelia after injury [3]. These epithelial stem/progenitor cells can generate lung organoids that provide a powerful platform for the study of human lung development and respiratory diseases and have therefore attracted intense interest from researchers and physicians. Several reviews on organoids are available [4–7], but in this review, we focus on organoid studies in the alveolar space, where damage is usually associated with difficulties in gas exchange or even lethal outcomes.

## 2. Stem/Progenitor Cells That Generate Alveolar Epithelia

### 2.1. Endogenous Stem/Progenitor Cells

The alveolar surfaces of both humans and mice are lined by alveolar type 1 (AT1) epithelial cells and alveolar type 2 (AT2) cells. AT1 cells cover the great majority of gas exchange surfaces and are extremely thin to facilitate gas diffusion [8]. AT2 cells are secretory epithelial cells that reduce surface tension and limit bacterial growth in alveoli [9]. During mouse lung development, AT1 and AT2 cells are generated directly from a bipotent progenitor [10, 11]. It remains to be determined whether or not a similar bipotent progenitor population exists in the human fetal lung; however, cells expressing both an AT2 cell marker (surfactant protein C (Sftpc)) and an AT1 cell marker (podoplanin) were observed in long-term cultured organoids derived from human embryonic stem cells [12]. In adults, AT2 cells were shown to give rise to AT1 cells; AT2 cells are therefore considered to be the facultative progenitor cells for alveolar epithelia in both humans and mice [13–15]. Animal models have been used to reveal the regenerative function of AT2 cells in adult alveolar epithelia. Studies are still underway to fully characterize the stem cells that replenish AT2 cells during steady state and after alveolar injury [15, 16]. Rare subsets of AT2 cells, including *α*6*β*4^+^ AT2 cells and Axin2^+^ AT2 cells, can generate AT2 cells after lung injury in mice [17, 18]. Zacharias et al. also described a Wnt-responsive alveolar epithelial progenitor in healthy human lungs [19]. In the bronchoalveolar duct junction, bronchioalveolar stem cells (BASCs), characterized by coexpression of secretoglobin family 1A member 1 (Scgb1a1, also known as CCSP, CCPBP, CC10, or CC16) and Sftpc, were demonstrated to be capable of repairing alveolar epithelia and distal airway epithelia [20, 21]. Basal cells, previously believed to be absent in this location, have been proposed to migrate to the alveolar region and generate AT2 cells after a viral infection in mice [22]. But in human lungs, the presence of basal cells is apparent in terminal bronchiole in addition to proximal airways [23]. In addition, AT1 cells, previously thought to be terminally differentiated, have been shown to exhibit plasticity potential to dedifferentiate into AT2 cells in a mouse model of partial pneumonectomy [24, 25]. Thus, injury-specific repair mechanisms may exist for rapid recovery of alveolar epithelia to secure the gas exchange function by accelerating AT2 cell regeneration.

### 2.2. Pluripotent Stem Cells

Stepwise approaches have been successfully established for the differentiation of embryonic stem cells (ESCs) into functional alveolar cells [26–29]. Induced pluripotent stem cells (iPSCs) are the product of adult somatic cells that are reprogrammed into an embryonic-like state, and their usage has become an effective strategy for developing patient-specific lung epithelial cells. Huang et al. reported an optimized method to generate FOXA2^+^NKX2.1^+^ progenitor cells from human definitive endoderm cells at an efficiency rate of 86% [30]. FOXA2^+^NKX2.1^+^ progenitor cells were shown to give rise to basal, Club, goblet, ciliated, AT1, and AT2 cells both *in vivo* and *in vitro* [30]. Gotoh et al. induced human iPSCs to form NKX2-1^+^ “ventralized” anterior foregut endoderm cells, from which cells expressing carboxypeptidase M (CPM) were sorted for 3D coculture with fetal human lung fibroblasts [31]. The resulting CPM^+^ organoids contained mostly AT2 cells, as well as some AT1, ciliated cells, and goblet cells, but not Club cells [31]. These iPSC-derived AT2 cells exhibit phenotypic properties similar to those of mature human AT2 cells, including lamellar body-like structures and surfactant protein expression [31]. The induction efficiency of AT2 cells was later substantially improved by preconditioning NKX2-1^+^ “ventralized” anterior foregut endoderm cells [32]. However, a relatively homogeneous population of AT2 and AT1 cells was generated from human iPSCs reprogrammed from fetal or neonatal lung fibroblasts [33]. Human iPSC-derived AT2 cells exhibit a self-renewal capacity and display immune responsiveness [32, 34]. These studies clearly showed that cell lineages produced from iPSC-derived organoids are tightly controlled by signaling pathways associated with organ development. Fibroblast growth factor (FGF) signaling was shown to promote the induction of anterior foregut endoderm into human lung organoids possessing both mesenchymal and lung epithelial cells, including primarily basal cells, as well as ciliated cells and AT2 and AT1 cells at a low abundance [35].

## 3. Development of Alveolar Organoids

**Figure 1:**
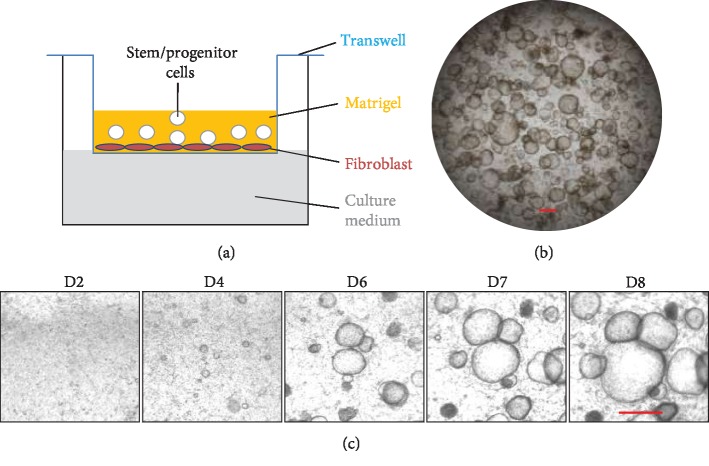
*In vitro* organoid culture of distal mouse lung stem/progenitor cells. (a) Distal lung stem/progenitor cells were mixed with Matrigel, loaded into Transwell filter inserts, and placed in 24-well culture plates containing culture medium. (b) Representative image of organoid culture on day 8 after seeding. (c) Growth period of organoids *in vitro*. Scale bar: 500 *μ*m.

Before alveolar organoid culture was established, it was realized that feeder cells (usually fibroblasts) are critical for the *in vitro* growth of BASCs [20]. One year after the first report on intestinal epithelial stem cell-derived organoids, McQualter et al. successfully established an organoid culture method for bulk lung epithelial cells, including stem/progenitor cells in the presence of fractionated primary mouse lung stromal cells in transwells sitting on a 24-well plate [36, 37]. These lung stem/progenitor cells formed organoids within 1 month. Organoid culture was later optimized by replacing primary mouse lung stromal cells with immortalized MLg mouse lung fibroblasts (also known as CCL206), which overcame the difficulty of isolating primary mouse lung fibroblasts and shortened the length of organoid cultures to 1 week [38, 39] (Figure 1). However, not all lung fibroblast cell lines support organoid cultures of distal lung stem/progenitor cells. For example, CCL39, another mouse lung fibroblast cell line, does not support organoid culture of distal lung stem/progenitor cells. The ability of MLg cells to support distal lung stem/progenitors is reduced when they overgrow. These findings suggest that the secretory properties of supportive fibroblasts are critical for successful organoid culture of endogenous lung stem/progenitor cells. Conditioned medium harvested from fibroblast cultures is less supportive for the organoid culture of distal lung stem/progenitor cells, probably because the concentration of key growth factors is insufficient. Distal lung stem/progenitor cells generate organoids with low colony-forming ability when stromal cells are replaced by high concentrations of FGF10 and hepatocyte growth factor, suggesting that other growth factors are needed for alveolar organoid development. In contrast, isolated human distal lung epithelial cells, usually including basal cells, generate organoids in the absence of mesenchymal support [40]. Human PSC-derived AT2 cells form 3D alveolospheres without the need for feeder cells [34]. These data suggest that autocrine growth factors play an essential role for such cells.

Indeed, *in vitro* organoid cultures provide a useful platform to reveal the interactions between stem/progenitor cells and niche cells in the lung. Lee et al. reported that lung endothelial cells also support BASC organoid cultures [41]. Mouse AT2 cells can form organoids in the presence of CD45^+^F4/80^+^ mouse macrophages [42]. Organoid culture assay allows us to readily address the interactions between alveolar stem/progenitor cells and other structural and immune cells in the lung.

In addition to this *in vitro* assay, organoid culture of distal lung epithelial stem/progenitor cells can be performed *ex vivo*. Epithelial spheroid structures form when a mixture of distal lung progenitor cells and Matrigel is subcutaneously injected into the back of mice. When grafted under the renal capsule, adult *α*6*β*4^+^ AT2 subset cells differentiate and regenerate epithelial structures within 1 week [17]. Longer organoid culture of human PSC-derived lung epithelial progenitor cells under the renal capsule can even generate branching structures [43]. An obvious benefit of *ex vivo* organoid culture is that a capillary network usually develops over the spheroid structures, which is not seen in *in vitro* assays.

Human distal EpCAM^+^ epithelial cells, including AT2 cells, can be isolated from peripheral lung tissue specimens by magnetic bead sorting (MACS) [44]. Coculture of these EpCAM^+^ cells with MRC5 human lung fibroblasts in Matrigel resulted in the formation of organoids that allows airway differentiation but not alveolar differentiation [44]. Alveolar differentiation was promoted by inhibiting TGF-*β* receptor signaling in organoids derived from human distal airway *Δ*Np63^+^TTF-1^+^ stem cells [40]. Human *Δ*Np63^+^TTF-1^+^ stem cells were also capable of differentiating into airway ciliated and Club cells in the presence of FGF10 and a *γ*-secretase inhibitor [40]. Human AT2 cells are typically isolated from dissociated human lungs through fluorescence-activated cell sorting (FACS) or MACS with the use of a monoclonal antibody, HTII-280, which is specific to human AT2 cells [4, 45]. A Wnt-responsive alveolar epithelial progenitor expressing transmembrane-4 L-six family member-1 (TM4SF1) within the human AT2 cell population, isolated from the distal lung by FACS (HTII-280^+^/TM4SF1^+^/EpCAM^+^), generated 3D alveolar organoids in the presence of MRC5 fibroblast cells that contain both AT2 and AT1 cells [19].

In addition to surgically removed human lung tissue, organoids can be generated from the culture of human lung epithelial cells collected from bronchoalveolar lavage fluid (BALF) or rectal biopsies for certain purposes [46]. Isolated human lung stem/progenitor cells may be cryopreserved and thawed later for organoid culture [47]. Human ESC/iPSC lines can be generated from patient samples, for example, dermal fibroblasts, for the development of alveolar organoids under appropriate conditions [34].

## 4. Analysis of Alveolar Organoids

The functional analysis of organoids usually includes several aspects. First, the colony-forming ability of distal lung stem/progenitor cells is evaluated in *in vitro* organoid assays based on the percentage of the number of colonies to the number of plated stem/progenitor cells. The colony-forming efficiency (CFE) of mouse AT2 cells ranges from 0.5 to 2% [48, 49] because of variations in culture conditions, whereas the CFE of distal airway stem/progenitor cells ranges from 0.5 to 4% [39, 41]. The CFE of human AT2 cells ranges from 2 to 8% [19, 47]. To investigate the self-renewal potential of stem cells, colonies are broken down into single cells followed by replating in Matrigel for organoid cultures. After 2-3 passages of the organoid cultures, the self-renewal capacity of stem/progenitor cells can be evaluated by comparing CFE among passages. Second, the average size of a colony, measured via the diameter or the surface area of individual colony, reflects the proliferation potential of the seeded stem/progenitor cells or swelling induced by water channels on the cell surface allowing the evaluation of the membrane permeability and secretion potential of the cells in the organoids [46, 48]. Culture medium can be supplemented with BrdU to allow for BrdU incorporation analysis in organoid end cultures [48]. Alternatively, to further evaluate the proliferation of stem/progenitor cells, organoid cultures can be fixed, embedded, and sectioned for Ki67 immunostaining [48]. Lastly, differences in the differentiation potential of stem/progenitor cells are evaluated by immunostaining sections for AT2 (pro-SPC) and AT1 (T1*α*, aquaporin 5) cells. Organoid cultures can also be harvested to analyze these markers at the transcriptional level via quantitative polymerase chain reaction. Transcriptome analysis in bulk or at a single cell level can also be conducted for alveolar organoids. In addition, organoid cultures can be processed for electron microscopic analysis to visualize the general structures of the individual organoids established in *in vitro* or *ex vivo* assays.

## 5. Organoid Modeling of Idiopathic Pulmonary Fibrosis

IPF is characterized by progressive fibrotic scarring in the lung tissue surrounding the air sacs, which ultimately leads to dyspnea. TGF-*β* is upregulated and activated in IPF and modulates fibroblast phenotype and function in the lung. Although the bleomycin-induced mouse model and others have some gross similarities to human IPF, they fail to faithfully reproduce the pathophysiology of the disease [50]. A number of candidate drugs identified in preclinical animal studies failed in human clinical trials, leaving only two FDA-approved drugs for IPF treatment: pirfenidone and nintedanib. Wilkinson et al. generated a model of the progressive scarring that resembles human IPF by treating induced human PSC-derived mesenchymal cell organoids with TGF-*β* [51]. Human PSCs have been shown to generate functional alveolar epithelial cells [34]. By using CRISPR/Cas9 to introduce frameshift mutations in Hermansky-Pudlak syndrome (HPS) genes, human ESC-derived lung organoids show fibrotic changes, thus providing a platform to identify pathogenic mechanisms of IPF that are likely clinically relevant *in vitro* [52]. Using 3D lung organoids from patients with IPF, Surolia et al. observed that inhibiting the assembly of vimentin intermediate filaments reduces the invasiveness of lung fibroblasts in the majority of the subjects tested [53]. In addition, organoid assays provide an opportunity to evaluate functional alterations in mesenchymal niches in IPF, which is related to the proliferative potential of distal lung progenitor cells [54]. The development of 3D organoid models has created systems capable of emulating human distal lung structures, functions, and cell and matrix interactions, enabling preclinical antifibrotic drug testing [55].

Disrupted distal airway and alveolar epithelia are normally observed in patients suffering from IPF [3]. Endogenous lung epithelial stem/progenitor cell-derived organoid assays provide a unique platform for understanding the mechanisms of adult distal lung diseases. In the IPF mouse disease model, intratracheal instillation of bleomycin results in loss of AT2 progenitor cells [56]. The surviving AT2 cells proliferate and differentiate to replenish the alveolar epithelium. The development of fibrotic obliteration of lung alveoli is believed to be driven by incomplete repair of injured alveolar epithelia, which is closely related to the repair capacity of the surviving stem/progenitor cells, including AT2 cells [57, 58]. In addition, as the surfactant-producing cells of the alveoli, AT2 cells secrete surfactant to maintain surface tension and alveolar patency. Mutations in these surfactants are associated with pathogenesis in some familial and sporadic forms of IPF in humans and IPF animal models [59–62]. Therefore, organoid cultures of AT2 cells and/or other alveolar stem/progenitor cells from IPF patients can be used to test the reparative and/or secretory function of these stem/progenitor cells. Drug treatment can be optimized by screening cells that promote the regenerative capacity of alveolar stem/progenitor cells and restore surfactant secretion to the normal level.

## 6. Organoid Modeling of Pulmonary Tuberculosis

Since its discovery by Robert Koch in 1832, *Mycobacterium tuberculosis* (MTB) remains a great health threat to the global population, particularly in Southeast Asian and some African countries [63]. Nearly ten million new MTB infections have been recognized annually during the past 5 years according to a WHO TB report. Drug-resistant strains and coinfection with HIV are challenging the End TB Strategy by 2035 proposed by the WHO [64]. Animal models that have been used to investigate TB pathologies and drug screening possess several obvious faults. First, the facility that houses animals with MTB infections is usually expensive, which hinders its extensive usage in TB research. In addition, the animals are not natural hosts for MTB, so they only partially mimic TB clinical signs, characteristic pathological lesions (granuloma formation and lung cavitation), and immunological indices [65, 66]. Consequently, organoids are emerging as a promising technology to study host-MTB interactions in a dish. Human lung organoids have been successfully established using different technologies [46, 67, 68]. The obvious advantage of human lung organoids relies on their spatial organization and the heterogeneity of their cellular components. MTB infections of alveolar organoids not only allow for the inclusion of very early-stage MTB, which is difficult to follow in animal models, but also overcome species differences [68]. In this case, human alveolar organoids can be used to study the direct interactions between MTB and lung epithelium by injecting MTB into developed organoids, which usually grow up to 500 *μ*m in diameter. Alternatively, immune cells, including macrophages, can be introduced into the organoid structure to mimic the *in vivo* complexity of the immune response.

## 7. Organoid Modeling of Respiratory Viral Infections

Viral infections in the distal lung have been implicated in the progression of pneumonia to acute respiratory distress syndrome. Respiratory viruses, including COVID-19, target lung epithelial cells, including AT2 cells [2]. Influenza viruses target AT2 and AT1 cells after intratracheal infection in mouse models [69]. Studies of human respiratory infections have been limited by the paucity of functional models that mimic *in vivo* physiology and pathophysiology [70]. The establishment of *in vitro* organoid cultures offers remarkable model systems to study disease pathogenesis and host-virus interactions. Porotto et al. observed the spread of human parainfluenza virus 3 (HPIV3) in 3D lung organoids derived from hPSCs and infected AT2 cells in the organoids [71]. Consistent with the clinical observations for HPIV3 infection, changes in tissue integrity and shedding of infected cells into the lumen were not observed in these organoids [71]. A morphological analysis of respiratory syncytial virus- (RSV-) infected human lung organoids derived from hPSCs revealed massive epithelial alterations that recapitulate *in vivo* pathologies, including apical extrusion of infected cells, cytoskeletal rearrangement, and syncytia formation [43]. RSV replicates readily in human airway organoids, and the entry of RSV into airway organoids can be prevented by palivizumab, an antibody that blocks RSV–cell fusion [72]. Palivizumab and other antiviral drugs may also be evaluated in alveolar organoids for their antiviral capacity. Similar organoids have been established to rapidly assess the infectivity of emerging influenza viruses to humans [73, 74]. Therefore, such organoid technology can be applied to study host-pathogen interactions in a range of lung pathogens.

## 8. Conclusions and Perspectives

**Figure 2:**
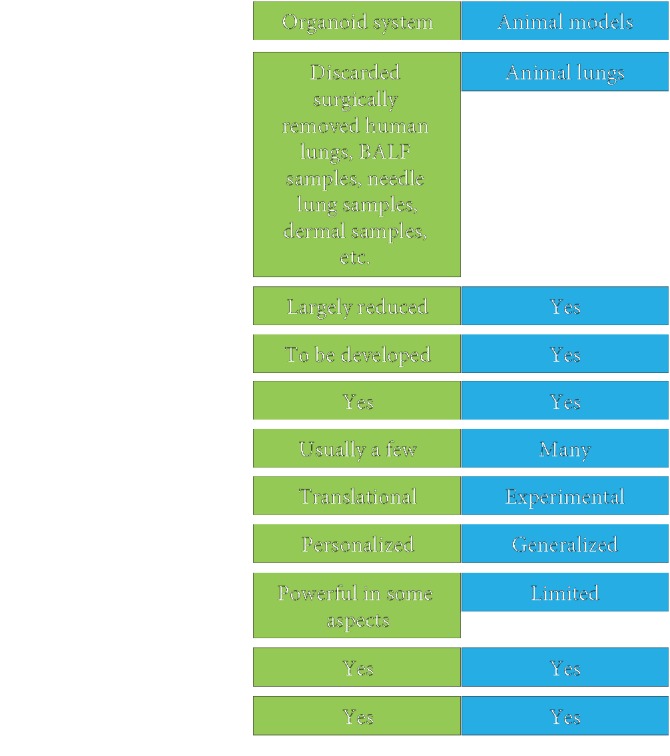
Comparison of the organoid system and animal models.

Disruptions to alveolar epithelia are associated with various refractory respiratory diseases. Organoid technology serves as a new pathological model to investigate cell-cell crosstalk and host-pathogen interactions and is a powerful platform for modeling human lung diseases and for drug screening and toxicity assays. This tool could replace some animal experiments, thereby minimizing animal use in respiratory research (Figure 2). A bank of human lung organoids could be established for cell or gene therapy. Generating personalized organoids would also open novel avenues for research into individual responses to therapies and thus also for the implementation of personalized medicine. However, there are still some limitations to using organoid technology to model distal lung diseases. Distinct from other organs, lungs inflate and deflate during gas exchange, generating a force that is currently hard to model in organoids. Most importantly, there is still a lack of established *in vitro* alveolar organoids with developed vasculature, although blood vessels grow over implanted organoids *ex vivo*. Undoubtedly, future optimized organoid technology will continue to significantly advance fundamental, therapeutic, and clinical research into respiratory diseases for decades to come.

## Abbreviations

AT2: Alveolar type 2 cells
BASC: Bronchioalveolar stem cell
IPF: Idiopathic pulmonary fibrosis
RSV: Respiratory syncytial virus
BALF: Bronchoalveolar lavage fluid
CFE: Colony-forming efficiency
MTB: *Mycobacterium tuberculosis*
ESCs: Embryonic stem cells
iPSCs: Induced pluripotent stem cells.
